# Evolving Therapeutic Paradigms in Pediatric Hypophosphatasia: From Survival-Driven Care to Integrated Precision Management

**DOI:** 10.3390/ijms27156921

**Published:** 2026-08-01

**Authors:** Alexandru Florescu, Teodora Cristina Vintilă, Ioana Vasiliu, Oana Viola Bădulescu, Ancuța Lupu, Iris Bararu-Bojan, Vasile Valeriu Lupu, Bianca Simionescu, Cristina Grosu, Andreea Iațentiuc, Ingrith Miron, Otilia Elena Frăsinariu

**Affiliations:** 1 Grigore T. Popa University of Medicine and Pharmacy, 700115 Iași, Romania; alexandru-florin.florescu@umfiasi.ro (A.F.); ioana_medgen@yahoo.com (I.V.); frasinariu.otilia@umfiasi.ro (O.E.F.); 2 Endocrinology Department, Saint Spiridon County Hospital, 700111 Iași, Romania; 3 Mother and Child Department, Iuliu Hatieganu University of Medicine and Pharmacy, 400012 Cluj-Napoca, Romania

**Keywords:** hypophosphatasia, *ALPL* gene, tissue-specific alkaline phosphatase, enzyme replacement therapy, newborn screening

## Abstract

Hypophosphatasia (HPP) encompasses a group of inherited metabolic bone disorders characterized by defective skeletal mineralization and variable clinical severity in childhood. Substantial allelic heterogeneity contributes to a broad pediatric clinical spectrum, ranging from life-threatening perinatal disease to milder phenotypes characterized by chronic functional impairment. Historically, management relied primarily on supportive interventions aimed at sustaining survival, without modifying the underlying enzymatic defect. The introduction of enzyme replacement therapy (ERT) with asfotase alfa has fundamentally altered the natural history of pediatric HPP by supplementing deficient alkaline phosphatase activity at sites of active mineralization, thereby improving skeletal integrity, enhancing survival in severe forms, and supporting long-term functional gains. This therapeutic shift has redirected clinical priorities from survival alone toward sustained functional development and health-related quality of life. Nevertheless, variability in disease expression and therapeutic response persists, reflecting both diagnostic timing and the molecular heterogeneity of *ALPL* variants, whose phenotypic consequences cannot be predicted with complete certainty. Growing recognition of the importance of early diagnosis has prompted exploratory efforts toward systematic identification strategies, including neonatal screening initiatives reported in selected populations, which suggest the potential for earlier therapeutic intervention during active skeletal development. Together, these considerations highlight pediatric HPP as a model of precision-oriented management in rare metabolic bone disease, where timely diagnosis and targeted enzyme replacement must be aligned with long-term, multidisciplinary care to optimize outcomes.

## 1. Introduction

Hypophosphatasia (HPP) is a rare, inherited metabolic disorder caused by loss-of-function mutations in the *ALPL* gene, which encodes tissue-nonspecific alkaline phosphatase (TNSALP) [1]. The deficiency of TNSALP leads to the accumulation of its physiological substrates: inorganic pyrophosphate (PPi), pyridoxal-5-phosphate (PLP), and phosphoethanolamine (PEA). PPi represents an inhibitor of mineralization and the accumulation of this can block hydroxyapatite crystal formation within the skeletal matrix causing rickets during growth period or osteomalacia in adult life [2]. PLP, the active form of vitamin B6, cannot cross the blood–brain barrier in its phosphorylated form; in HPP, elevated serum PLP reflects systemic accumulation and is associated with seizures, particularly in neonates with severe forms of the disease [3,4]. PEA, although less well understood, serves as an additional biomarker of disease activity and contributes to the biochemical profile of HPP [1]. The combined accumulation of these substrates not only reflects the severity of enzymatic deficiency but also underscores the multisystemic nature of the disease and the need for a targeted therapeutic approach.

The phenotypic expression of HPP is remarkably diverse, a reflection of its genetic heterogeneity, with over 300 known *ALPL* mutations [5,6,7]. Despite reported genotype–phenotype correlations, clinical severity cannot be reliably predicted based on genotype alone, highlighting the complexity of molecular determinants underlying disease expression. Accordingly, HPP may be inherited in either an autosomal recessive or autosomal dominant manner, with the most severe phenotypes, including perinatal lethal and infantile forms, typically transmitted as autosomal recessive traits, whereas milder presentations may arise from either recessive or dominant inheritance. Clinically, the disease is classified based on the age of onset and whether dental manifestations occur in isolation or are accompanied by systemic features. This framework yields six major subtypes: perinatal, perinatal benign, infantile, childhood, adult, and odonto-HPP [8,9]. In odonto-HPP, premature loss of primary teeth may be the sole manifestation, whereas other forms involve a spectrum of complications including skeletal deformities, fractures, growth impairment or respiratory compromise.

Historically, treatment of HPP relied solely on symptomatic and supportive care, aimed at mitigating complications without addressing the underlying enzyme deficiency. However, the development and regulatory approval of enzyme replacement therapy (ERT) with asfotase alfa, a recombinant TNSALP fused to a bone-targeting deca-aspartate motif, has transformed the therapeutic landscape. By restoring extracellular ALP activity and reducing toxic substrate accumulation, asfotase alfa enables physiological mineralization, improves survival in severe pediatric forms, and offers meaningful gains in function and quality of life [10].

This review examines the therapeutic evolution of pediatric HPP, with a particular focus on the pharmacological transition from symptomatic management to ERT with asfotase alfa. It traces how supportive and symptomatic interventions, once the mainstay of care, have been redefined as complementary strategies aimed at optimizing functional outcomes and quality of life in the era of targeted enzymatic correction. Furthermore, it addresses the ongoing need for harmonized diagnostic protocols and early recognition strategies to reduce therapeutic delay. The aim of this review is to integrate current genetic, molecular and clinical evidence on pediatric HPP in order to provide a comprehensive overview of the evolving therapeutic paradigm, with particular emphasis on how advances in disease biology have reshaped diagnostic strategies, therapeutic individualization, and long-term management within the framework of precision medicine.

## 2. Methodology

**Figure 1:**
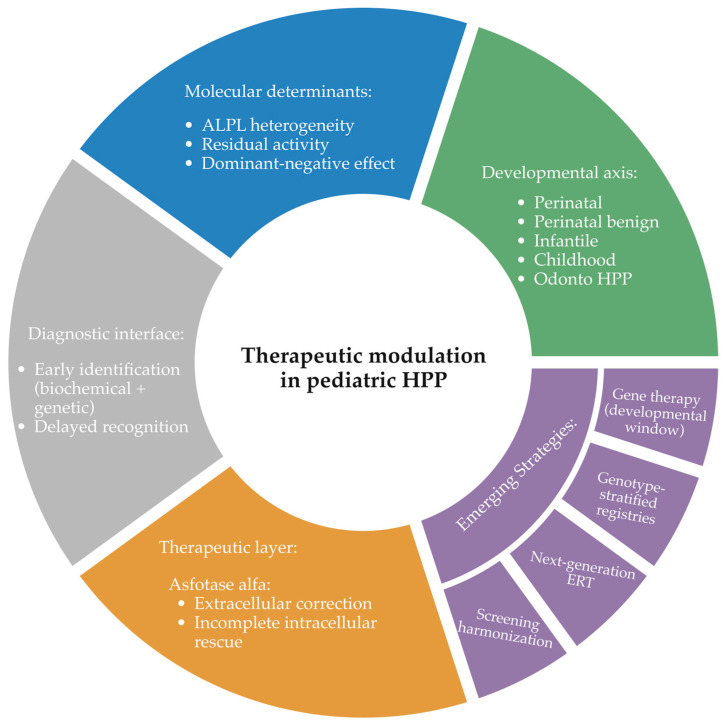
Conceptual framework illustrating the interdependence between developmental timing, diagnostic pathways, therapeutic strategies, and underlying molecular determinants in pediatric hypophosphatasia.

This narrative review was based on a comprehensive search of peer-reviewed literature published over the past two decades. Relevant articles, including clinical trials, registry reports, consensus guidelines and high impact reviews, with particular emphasis on pediatric cohorts were identified via PubMed, Scopus, and Google Scholar. The search strategy combined the keywords “hypophosphatasia”, “asfotase alfa”, “pediatric”, “enzyme replacement therapy”, “symptomatic management”, “diagnosis”, and “screening”. The retrieved literature was critically evaluated and synthesized with the aim of integrating molecular, genetic and clinical evidence. The interrelated molecular, developmental, diagnostic, and therapeutic dimensions are conceptually summarized in Figure 1.

## 3. The Contemporary Role of Symptom-Directed Management in the Ert Era

The advent of ERT has fundamentally transformed the therapeutic landscape of HPP, shifting the focus from palliative, symptom-based strategies toward targeted molecular correction. However, this paradigm shift does not imply the obsolescence of traditional supportive care. On the contrary, the role of symptom-directed management has evolved rather than disappeared, assuming a complementary and rehabilitative function within an integrated, long-term treatment framework. In pediatric HPP, where chronic pain, muscular weakness, and dental manifestations may persist even under effective enzyme replacement, supportive interventions continue to play a vital role in sustaining mobility, growth, and quality of life [11]. Although ERT addresses the biochemical defect underlying impaired mineralization, it cannot alone reverse all structural or functional consequences of the disease, particularly in patients with delayed diagnosis or residual deformities. In this context, historically supportive measures, when applied thoughtfully and in coordination with ERT, remain indispensable components of comprehensive care.

### 3.1. Physical Therapy and Exercise

For children living with HPP, maintaining strength, mobility, and endurance represents a central aspect of daily functioning and perceived well-being. Even when skeletal deformities are controlled through ERT, residual muscular weakness, stiffness, and early fatigability often persist [12]. These limitations highlight the need for structured physiotherapy and tailored exercise programs that address the broader neuromuscular dimension of the disease.

When implemented under expert supervision, physiotherapy and exercise interventions are regarded as safe, empirically beneficial, and free from significant adverse effects. Early initiation of physical rehabilitation facilitates neuromuscular adaptation, enhances postural control, and supports the acquisition of motor milestones, particularly in children who begin ERT after the onset of hypotonia or skeletal deformity [13]. Exercise prescriptions should be individualized according to disease severity, skeletal stability, and fatigue tolerance. Given the frequent complaints of muscular tension, particularly in the cervical and shoulder regions, relaxation and stretching techniques may serve as useful adjuncts, although standardized protocols are yet to be established. Equally important is the inclusion of home-based programs, which promote consistency, autonomy, and family involvement in long-term rehabilitation [14].

Beyond their musculoskeletal benefits, structured physical interventions also yield psychosocial advantages. Participation in physical activity fosters self-confidence, emotional resilience, and social engagement, factors that significantly contribute to perceived quality of life in pediatric HPP [15]. Within the framework of precision medicine, physiotherapy thus remains an essential complement to ERT, bridging biochemical correction with functional recovery and developmental reintegration. Nevertheless, systematic evaluation of exercise outcomes remains scarce, and well-designed prospective studies are warranted to define optimal rehabilitation protocols in pediatric HPP.

### 3.2. Orthotics

Orthotic management represents an important adjunctive component of care in pediatric HPP, addressing the mechanical consequences of impaired skeletal mineralization and low muscular tone. A broad spectrum of orthotic devices, from simple corrective insoles to more complex functional or stabilizing braces, can be used to improve postural alignment, redistribute mechanical load, and enhance joint stability during growth [16].

Beyond providing passive external support, orthoses play an active biomechanical role by guiding gradual alignment and promoting symmetrical weight-bearing patterns. In this context, early and individualized orthotic intervention can help prevent the progression of deformities and, in some cases, support their partial correction through growth-mediated remodeling [17]. Such proactive management is particularly relevant given the high prevalence of reduced muscular stabilization in children with HPP, predisposing not only to lower-limb deformities such as plano-valgus feet and genu valgum but also to axial malalignments, including scoliosis [16]. Orthopedic bracing, when applied judiciously and integrated within a multidisciplinary rehabilitation program, can therefore serve both preventive and corrective purposes. By enhancing postural control and reducing compensatory strain, orthotics contribute not only to improved gait mechanics but also to overall functional endurance and comfort. The selection and adjustment of devices should be performed under specialist supervision, ideally in coordination with physiotherapists familiar with metabolic bone disease, to ensure optimal fit, compliance, and dynamic adaptation as the child grows [16,18].

Although orthotic care does not target the underlying enzymatic defect, it remains a central element of symptom-directed management in pediatric HPP and plays a complementary role alongside ERT. In the modern therapeutic paradigm, orthotic care extends beyond structural correction; it functions as an active bridge between enzyme replacement and functional rehabilitation. By restoring biomechanical alignment and reducing compensatory strain, it reinforces the long-term benefits of enzyme therapy, supports musculoskeletal resilience, and enhances children’s capacity for independent and meaningful participation in daily life [18].

### 3.3. Dental and Oral Care

Dental care represents a distinctive yet often underappreciated aspect of symptom-directed management in pediatric HPP. Even in the era of ERT, oral manifestations remain clinically relevant, particularly in children with odonto-HPP or those with partial systemic involvement. The premature exfoliation of primary teeth continues to serve as both a diagnostic hallmark and a therapeutic challenge with direct implications for long-term oral function and quality of life [19].

Premature exfoliation of deciduous teeth most commonly affects the anterior dentition, typically beginning with the mandibular primary incisors in milder forms of the disease, while in moderate phenotypes the deciduous molars may also be involved [20]. Tooth mobility is often the earliest clinical manifestation and frequently represents the reason families seek medical advice, being most commonly identified by a general dentist and prompting referral for specialist evaluation. From a pathophysiological perspective, histological analyses suggest that absent or severely deficient cementum, secondary to low TNSALP activity, constitutes a key mechanism underlying early tooth loss. In line with this observation, comparative studies of teeth from children with HPP and healthy controls have demonstrated alterations in both acellular and cellular cementum, whereas dentin mineral content appears to be largely preserved [21,22]. Beyond premature tooth exfoliation, HPP is associated with a broader spectrum of dental abnormalities affecting tooth morphology, structure, and color. Structural defects such as enamel and dentin hypoplasia, thin and shortened roots, enlarged pulp chambers, and an increased susceptibility to dental caries are commonly reported. Defective mineralization of enamel and dentin may further contribute to alterations in tooth color, ranging from dark yellow to brown [23,24]. Importantly, the severity and extent of these dental abnormalities often parallel overall disease severity, with more pronounced defects observed in more severe phenotypes [25].

Modern management of oral complications in HPP emphasizes prevention, surveillance, and function-oriented intervention rather than reactive treatment. Regular dental evaluations allow for the early detection of mobility, delayed eruption of permanent teeth and characteristic radiographic abnormalities. The diagnostic process of odonto-HPP relies on careful clinical assessment of atypical tooth mobility relative to age, complemented by radiographic evaluation using orthopantomography and periapical intraoral radiographs, which may reveal molars with a shell-like appearance due to enlarged pulp chambers and thin dentin walls [25]. Clinicians should avoid unnecessary extractions and prioritize conservative approaches aimed at maintaining primary dentition for as long as feasible, as early tooth loss can impair mastication, speech, and craniofacial development. Preventive strategies such as fluoride varnish applications, meticulous oral hygiene reinforcement help minimize secondary complications like enamel attrition or gingival irritation [26,27].

In patients already receiving ERT, dental monitoring remains indispensable for tracking structural adaptation within the alveolar bone and evaluating the stability of both primary and permanent dentition. Preliminary observations suggest that early initiation of ERT may improve root anchorage and reduce the incidence of premature exfoliation; however, long-term data remain limited. Until such evidence becomes available, structured dental follow-up remains a cornerstone of comprehensive pediatric HPP management [28].

**Figure 2:**
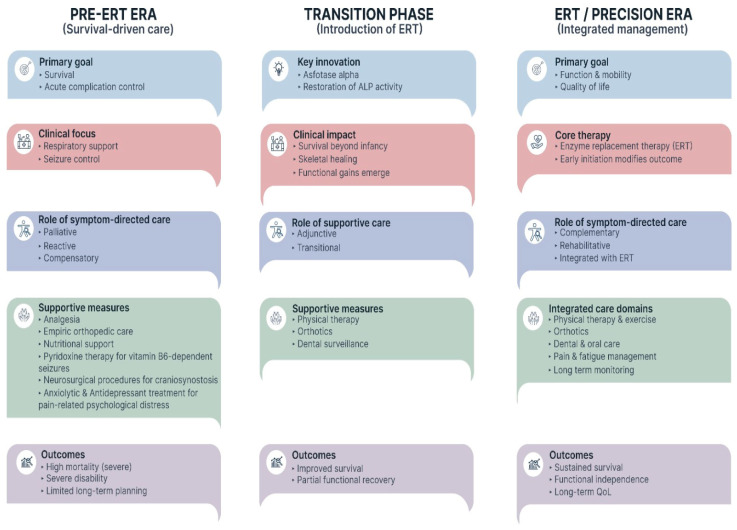
Evolution of therapeutic paradigms in pediatric HPP across the pre-ERT and ERT eras. Schematic overview of the transition from survival-driven supportive care in the pre-ERT era, through the introduction of enzyme replacement therapy, to the current ERT/precision era characterized by integrated, multidisciplinary management. The figure summarizes changes in therapeutic goals, clinical focus, and the role of symptom-directed care across these stages.

Viewed in this broader context, oral and dental manifestations represent one component of a wider reconfiguration of care priorities in pediatric HPP, in which survival-driven supportive management has evolved into coordinated, precision-oriented strategies in the ERT era as schematically summarized in Figure 2.

## 4. Ert in Pediatric HPP-Clinical Practice and Challenges

### 4.1. Development and Translational Pathway of Asfotase Alfa in Pediatric HPP

The development of ERT for HPP marks a pivotal advance in the translational management of rare metabolic bone disorders. Early therapeutic attempts, including infusions of placental alkaline phosphatase (PLAP) and mesenchymal stem cell transplantation, failed to produce meaningful or sustained clinical benefit [29,30]. Interestingly, reports of transient improvement in women with mild HPP during pregnancy, linked to the physiological activity of placental ALP, provided an important biological clue [31]. These observations suggested that simple enzyme supplementation was insufficient; what was required was a bioactive enzyme capable of anchoring to mineralizing tissue and reproducing the physiological actions of TNSALP at the site of pathology.

This concept found its first rigorous experimental confirmation in preclinical studies using *Akp2*^−^/^−^ knockout mice, an established model for the perinatal lethal form of HPP. In 2008, Millán and colleagues provided definitive proof of principle that systemic administration of a bioengineered soluble form of human TNSALP could restore skeletal mineralization and prevent lethality in these animals [32,33]. The recombinant construct, later designated asfotase alfa, was designed through a rational fusion strategy that linked the catalytic domain of TNSALP to the Fc region of human IgG, improving molecular stability and circulatory persistence. To achieve tissue-specific delivery, a deca-aspartate (D_10_) motif was incorporated, conferring high affinity for hydroxyapatite and enabling targeted deposition at mineralizing surfaces. [33,34,35].

Following the success of preclinical studies in *Akp2*^−^/^−^ mice, which demonstrated complete rescue of skeletal mineralization and survival, the translation of asfotase alfa into human therapy represented not merely a test of efficacy but a validation of the central biochemical hypothesis underlying enzyme replacement in HPP [34].

Early-phase clinical studies in infants and young children with perinatal and infantile-onset HPP provided pivotal translational evidence supporting the therapeutic concept of targeted enzyme restoration. Patients receiving subcutaneous asfotase alfa exhibited radiographic improvement of rickets-like skeletal lesions and progressive gains in motor function, growth, and respiratory stability, outcomes that collectively reflected reactivation of physiological mineralization processes. Longitudinal extensions of pivotal trials, together with real-world registry analyses, consolidated these findings and highlighted the sustained durability of therapeutic benefit [8,36,37]. Children treated in early infancy not only survived but maintained gains in motor independence and functional endurance over several years of follow-up. Radiologic evaluations demonstrated progressive cortical thickening, while biochemical parameters remained within stable ranges, reflecting ongoing enzymatic activity and controlled substrate metabolism [38,39,40]. Beyond objective endpoints, caregivers frequently reported improved stamina, participation in daily activities, and overall psychosocial well-being, emphasizing the multidimensional impact of enzyme restoration on both patients and families [41].

### 4.2. Clinical Implementation and Outcome Assessment of Asfotase Alfa in Pediatric HPP

Following its translation from molecular design to clinical use, asfotase alfa has reshaped the therapeutic approach to pediatric HPP. This recombinant TNSALP, engineered with a bone-targeting deca-aspartate motif, has transformed a condition once managed through symptomatic and palliative measures into one amenable to targeted, disease-modifying intervention [42,43]. Yet as clinical experience with the drug has expanded, several key challenges have emerged, not only in defining optimal dosing regimens, treatment initiation thresholds, and long-term monitoring strategies, but also in achieving timely and accurate diagnosis, particularly among children with non-classical or attenuated phenotypes. The expanding clinical spectrum of pediatric HPP has revealed diagnostic gray zones in which biochemical markers may be inconclusive and radiographic changes subtle or absent, delaying recognition and therapeutic access. Initially developed for the most severe perinatal and infantile forms, asfotase alfa is now being used across a broader range of pediatric presentations, prompting ongoing debate regarding treatment eligibility, individualized response assessment, and the balance between early intervention and clinical necessity [44,45,46]. These issues collectively underscore the need for harmonized diagnostic criteria and evidence-based therapeutic algorithms capable of guiding personalized management in this heterogeneous disorder.

The rationale behind asfotase alfa lies in its ability to restore extracellular ALP activity, thereby clearing the pathological accumulation of inorganic pyrophosphate and pyridoxal-5′-phosphate, two substrates that interfere with bone mineralization and neuromuscular function [47]. Although the skeletal manifestations of HPP have historically dominated therapeutic considerations, recent insights underscore the multisystemic burden of the disease, which may justify treatment even in the absence of overt rickets. Elevated PLP, for instance, has been linked to neuromuscular dysfunction, and anecdotal evidence points toward improvements in muscle strength, fatigue, and even behavioral symptoms following initiation of therapy [40].

Crucially, the decision to initiate asfotase alfa is no longer binary. The traditional divide between “severe” and “mild” forms has been eroded by the growing recognition that even children without florid radiological rickets may harbor functionally significant disease. The 2024 criteria proposed by the Hypophosphatasia International Working Group recommend a multidimensional diagnostic based on a combination of major and minor criteria, requiring either two major criteria or one major criterion plus two minor criteria for diagnosis. Major criteria include persistent low age-adjusted serum alkaline phosphatase activity, pathogenic *ALPL* variants, and premature exfoliation of primary teeth while minor criteria include short stature or linear growth failure over time, delayed motor milestones, craniosynostosis and B6 responsive seizures. This structured yet flexible approach underscores that functional burden, rather than rigid phenotypic labeling, should guide therapeutic decision making [48].

Emerging real-world data supports this broader perspective. Analyses from the Global HPP Registry have revealed that nearly one-third of children who ultimately benefit from asfotase alfa initially lacked overt skeletal findings. Instead, they presented with fatigue, motor delays, or biochemical abnormalities that, left untreated, risked cumulative and possibly irreversible damage [49]. These insights have fostered a paradigm shift toward earlier, preemptive intervention in select cases, particularly when quality of life or developmental trajectory is at stake. Yet, significant variability persists regarding timing and thresholds for initiating treatment. While there is consensus on the urgency of treating perinatal and infantile HPP, the optimal window for starting asfotase alfa in childhood-onset cases remains contested. Some clinicians advocate for early initiation upon biochemical progression or symptom emergence, whereas others prefer a conservative approach, monitoring for clearer signs of functional impairment. In the absence of validated predictive algorithms, composite burden scoring (combining functional assessments, biochemical trends, and patient-reported outcomes) may provide a useful tool for individualized decision-making [49,50].

Therapeutic dosing with asfotase alfa was initially guided by data from pivotal clinical trials that established regimens of 1 mg/kg administered subcutaneously six times weekly or 2 mg/kg three times weekly [35]. This approach has demonstrated consistent efficacy in preventing or reversing skeletal complications in severe pediatric forms. However, its application in heterogeneous real-world populations, particularly in non-lethal or childhood-onset HPP, requires a more individualized approach [50]. Importantly, no formal dosing de-escalation strategies have been validated, and attempts to reduce dose or administration frequency remain empirical, often driven by individual tolerability or parental preference. In clinical practice, a minority of centers have reported dose reductions in children with non-severe phenotypes or early clinical remission, but such strategies lack standardized protocols and are not supported by evidence-based guidelines. Further complicating management is the absence of predictive markers for sustained response or safe tapering. Until prospective studies clarify these issues, standard dosing recommendations should remain the default therapeutic framework [43,49].

A critical, yet underexplored, aspect of asfotase alfa therapy is the long-term follow-up of pediatric patients, a domain fraught with logistical, clinical, and interpretive challenges. The complexity of HPP follow-up lies not only in monitoring therapeutic efficacy but also in anticipating and managing evolving needs as children age, enter puberty, or transition into adult care. While existing literature outlines structured intervals for laboratory and radiological assessment (at baseline, 3 months, 12 months, and annually thereafter) there is no consensus on the integration of these data into treatment algorithms [51,52].

Within this broader follow-up framework, biochemical surveillance represents a central yet methodologically challenging component of long-term disease monitoring. Serial measurements typically include ALP, PLP, PTH, vitamin D, calcium, and urine PEA. However, ALP values are elevated during ERT (>3000 U/L), and PLP levels may be confounded by in vitro degradation unless ALP inhibitors like levamisole are used during sample handling. This necessitates technical rigor and awareness of preanalytical artifacts to prevent misinterpretation. Compounding this issue is the fact that low ALP activity, although a hallmark of HPP, is not exclusive to this condition. It may also occur in other clinical scenarios such as critical illness, recent surgery, massive transfusion, or various nutritional deficiencies including hypomagnesemia, zinc, or vitamin C deficiency. Magnesium, in particular, serves as a crucial cofactor for ALP activity, and its depletion may artificially lower enzyme activity, thereby mimicking HPP [53]. Additionally, gastrointestinal disorders such as untreated celiac disease have also been linked to suppressed ALP levels. These alternative explanations must be carefully ruled out to avoid diagnostic error and inappropriate treatment initiation [54,55,56]. Moreover, interpretation of ALP values requires strict consideration of age- and sex-specific normative data. In pediatric populations, where bone growth is dynamic, ALP levels may physiologically be up to four times higher than in adults [57].

These interpretive challenges have direct implications for therapeutic decision-making. In this context, the potential for both under- and overtreatment emerges as a central challenge in long-term management. Biochemical patterns suggestive of undertreatment, such as persistently elevated PLP or hypercalcemia, must therefore be interpreted alongside functional status and radiologic findings rather than in isolation. Conversely, overtreatment may manifest insidiously through ectopic calcifications or altered bone remodeling, particularly after growth plate closure [58]. In the absence of reliable biochemical predictors of optimal therapeutic intensity, clinical judgment integrating laboratory trends with functional performance and imaging remains essential.

Together with the chronic treatment burden associated with frequent injections, this uncertainty in response assessment may contribute to challenges in long-term treatment adherence. Voluntary discontinuation of asfotase alfa by patients or caregivers has been documented in the literature, most frequently driven by treatment fatigue, perceived lack of benefit, or logistical burden. Such interruptions, even in seemingly mild cases, have been associated with clinical deterioration, including pain exacerbation, rickets and reduced mobility [59,60]. The risk of worsening is not uniform and appears to depend on multiple factors, such as baseline disease severity, residual growth potential, and the duration of observation following treatment cessation.

Collectively, these challenges underscore that effective management of pediatric HPP in the ERT era extends beyond correct dosing and biochemical monitoring, necessitating a broader framework for evaluating therapeutic response and long-term benefit. Assessing the clinical response to asfotase alfa therapy therefore presents additional challenges. Early clinical studies, conducted in an era of high infant mortality, necessarily prioritized survival and respiratory stabilization, reflecting an outcome framework focused on immediate life preservation rather than long-term functional recovery [61,62]. As long-term survival has become achievable, contemporary investigations have progressively shifted toward functional performance, patient-centered outcomes and quality of life related measures. This evolution in outcome assessment represents an objective marker of the broader therapeutic paradigm shift in HPP, signaling a transition from life preservation toward durable functional recovery and participation in daily life. Within this framework, symptom-directed management is no longer ancillary, but emerges as an essential contributor to outcomes that now define therapeutic success in the ERT era. This shift is further illustrated in Table 1, which summarizes representative pediatric HPP studies and their primary outcome measures.

As the evaluation of therapeutic success in pediatric HPP has progressively shifted toward long-term function and quality of life, attention has also turned to children at the mildest end of the disease spectrum. This includes individuals with biochemical abnormalities or pathogenic *ALPL* variants who lack overt clinical manifestations at the time of assessment. The follow-up of asymptomatic pediatric HPP remains poorly defined, with no evidence-based guidelines to inform surveillance strategies. In current clinical practice, expert consensus generally favors structured longitudinal monitoring, most commonly through annual evaluations focused on growth, motor development, and the emergence of new symptoms. Although many of these children may remain clinically stable over extended periods, longitudinal observation is warranted given the potential for phenotypic evolution over time [49].

Taken together, these considerations highlight the complexity of implementing ERT in pediatric HPP. The key challenges discussed above are summarized in Table 2.

## 5. Diagnostic Delay as a Determinant of Ert Outcomes in Pediatric HPP

Delayed recognition of HPP remains a key factor influencing clinical outcomes in children, even in the era of ERT [66]. Although asfotase alfa has transformed the management of pediatric-onset HPP, its efficacy depends largely on the timing of initiation relative to the phase of skeletal development. Because the enzyme restores extracellular alkaline phosphatase activity at sites of active bone mineralization, the therapeutic effect is greatest when treatment begins before irreversible structural abnormalities have occurred [10,67]. In a pivotal multicenter trial, infants who started therapy before one year of age demonstrated markedly higher one-year survival rates and more complete radiographic recovery than those treated after prolonged disease progression [58]. Real-world data from the Global HPP Registry have since reinforced these findings, showing that earlier treatment correlates with fewer respiratory complications, reduced hospitalization rates, and more favorable motor development trajectories [11,68]. Although no study has directly quantified a pharmacologic loss of efficacy with delayed treatment, these clinical observations collectively support the presence of a developmental therapeutic window during active bone growth, a period in which enzymatic replacement yields the most pronounced and durable benefits.

Despite this well-established time dependency of therapeutic response, timely initiation of treatment is frequently compromised by diagnostic delay. Registry-based and patient-reported data indicate that the median age at first manifestation of HPP is approximately 7.2 months, whereas the median age at confirmed diagnosis approaches 20.4 months, corresponding to an average delay exceeding one year. Delays are particularly pronounced in children whose symptoms emerge after infancy, and in adults with retrospectively documented pediatric-onset disease, diagnostic latency may extend over several decades [69]. These findings illustrate that, even in the context of increased disease awareness and expanding access to genetic testing, pediatric-onset HPP continues to be frequently underrecognized or misattributed to more common skeletal or metabolic conditions [47,69,70]. As a consequence, the timing of diagnosis often does not coincide with symptom onset. Analysis of diagnostic patterns further reveals distinct age-related peaks, with patients most commonly diagnosed before 6 months, between 2 and 10 years, and after the age of 50 [69]. This distribution likely reflects the convergence of clinical burden and symptom severity at key developmental stages in early life, as well as the manifestation of cumulative or late complications in adulthood. In pediatric populations, more overt clinical signs and greater disease awareness in recent years may explain comparatively shorter diagnostic delays than those observed in historical adult cohorts. Nevertheless, even modest delays during sensitive periods of growth and development may result in lasting structural and functional consequences.

Several systemic factors contribute to this diagnostic gap. The heterogeneity of HPP manifestations, ranging from skeletal deformities and motor delay to isolated dental findings, complicates early recognition, particularly in attenuated or non-classical forms. Moreover, biochemical screening is hindered by variability in laboratory methods and reference intervals for ALP [69]. Many clinical laboratories fail to apply pediatric- and sex-adjusted reference ranges, leading to under recognition of HPP, even when the result is technically abnormal. Additionally, family history is often overlooked in the diagnostic process, despite the well-established heritability of *ALPL* mutation. These procedural shortcomings collectively hinder timely confirmation of HPP, which relies on the integration of clinical presentation, persistently low ALP activity, and, when available, supportive *ALPL* genotyping.

Taken together, these observations illustrate that the therapeutic transformation achieved through ERT has not been matched by an equivalent transformation in diagnostic and early management practices. While the advent of ERT has redefined the prognosis of pediatric HPP, delays in recognition continue to expose patients to prolonged periods of suboptimal or inappropriate care. This discrepancy between therapeutic progress and diagnostic implementation now defines a key tension within the evolving management paradigm in HPP and provides the natural framework for examining the patterns of inappropriate management and therapeutic missteps that frequently precede diagnosis.

### 5.1. Inappropriate Management and Therapeutic Missteps Prior to Diagnosis

Before the advent of ERT and the broader recognition of HPP as a distinct entity, many children were managed empirically for conditions with overlapping clinical features such as rickets or osteogenesis imperfecta. These diagnostic uncertainties often led to the application of therapeutic regimens that were well intentioned but pathophysiologically inappropriate. In the recent HPP Impact Patient Survey, the majority of children for whom treatment data were available had received calcium and vitamin D supplementation prior to diagnosis, reflecting the frequent misclassification of HPP as nutritional rickets or other forms of metabolic bone disease unresponsive to vitamin D therapy [69].

Although such supplementation may transiently improve biochemical parameters, it fails to address the underlying enzyme deficiency and, in some cases, can exacerbate hypercalcemia and hypercalciuria, increasing the risk of nephrocalcinosis and renal dysfunction. Paradoxically, once hypercalcemia developed, some children were subsequently advised to follow low-calcium diets highlighting the inconsistency of empirical management approaches [39,63]. Both supplementation and restriction, when implemented without recognition of the underlying ALP deficiency, risk further disturbing mineral homeostasis and delaying effective treatment. A smaller proportion of pediatric patients were exposed to bisphosphonates prior to diagnosis [11]. This finding, although numerically limited, is clinically relevant: bisphosphonates, which act by suppressing bone resorption, can further impair skeletal mineralization in HPP, where bone turnover is already pathologically reduced.

Reports from pediatric cohorts describe worsening radiographic appearance and increased fracture risk following antiresorptive therapy in undiagnosed HPP. The use of bisphosphonates prior to diagnosis therefore exemplifies how phenotypic overlap with osteogenesis imperfecta or other metabolic bone disorders can lead to therapeutic interventions that inadvertently aggravate disease pathology [71,72]. Symptom-directed management was also frequent among children with undiagnosed or delayed-diagnosed HPP. Analgesic therapy was commonly prescribed to alleviate musculoskeletal discomfort, reflecting the persistent pain burden characteristic of this population. In isolated cases, psychotropic medication, such as antidepressants or anxiolytics, was reported, underscoring the psychological strain and emotional distress associated with chronic pain and prolonged diagnostic uncertainty during childhood and adolescence [69]. In this setting of symptom-driven management, growth hormone therapy was occasionally attempted in children presenting with impaired linear growth before the recognition of underlying HPP. These interventions, undertaken under the assumption of idiopathic or endocrine growth deficiency, have not demonstrated measurable benefit, as the primary limitation to growth in HPP lies in defective skeletal mineralization rather than hormonal insufficiency [41]. Their inclusion among pre-diagnostic treatment attempts underscores the ongoing clinical uncertainty surrounding pediatric bone fragility when the biochemical signature of low alkaline phosphatase is overlooked.

**Figure 3:**
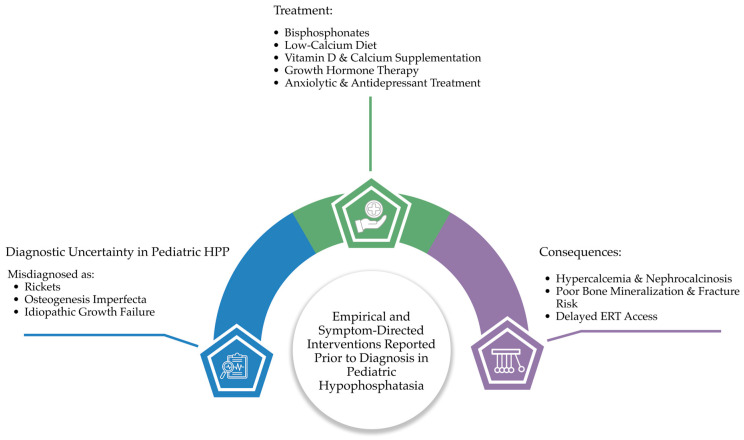
Summary of empiric and symptom-directed management strategies applied prior to diagnosis of pediatric HPP. Misclassification frequently led to vitamin D and calcium supplementation, low-calcium diets, bisphosphonate exposure, or other symptom-targeted therapies, reflecting diagnostic uncertainty and contributing to delayed enzyme replacement therapy. HPP hypophosphatasia.

Collectively, these empirical interventions illustrate the clinical consequences of delayed or missed diagnosis. Beyond their limited therapeutic value, they expose patients to unnecessary risks, contribute to avoidable healthcare costs, and, perhaps most importantly, defer access to ERT at a time when its benefits are most pronounced. As illustrated in Figure 3, the heterogeneity of these pre-diagnostic treatment pathways reflects persistent gaps in disease recognition and reinforces the need for structured diagnostic vigilance to ensure that pediatric HPP is identified and treated within its optimal therapeutic window.

### 5.2. Clinical and Psychosocial Consequences of Delayed or Absent Enzyme Replacement in Pediatric HPP

Although the biochemical and skeletal features of HPP are well delineated, the broader clinical implications of delayed recognition and postponed initiation of ERT remain substantial. Persistent musculoskeletal pain, functional impairment, and psychosocial distress observed in untreated or late-treated children demonstrate that the burden of HPP extends beyond the skeleton, reflecting the cumulative impact of chronic enzyme deficiency on growth, mobility, and quality of life. Within this context, timely initiation of asfotase alfa emerges not only as a determinant of skeletal recovery but also as a critical modifier of long-term functional and developmental outcomes.

#### 5.2.1. Chronic Pain

Chronic musculoskeletal pain is a frequent and often underrecognized manifestation of HPP, reflecting the cumulative structural consequences of impaired bone mineralization. Recent registry data and patient-reported studies have begun to clarify its prevalence and clinical relevance across the lifespan, with pain reported in approximately one fifth of pediatric patients and up to three quarters of adults [69]. The lower rates observed in children likely underestimate the true burden, as young patients may lack the capacity to verbalize or localize pain effectively. Furthermore, both parents and healthcare providers tend to underrate pain intensity in children, a bias well documented in pediatric pain literature, contributing to delays in adequate assessment and intervention [73,74].

In the HPP Impact Patient Survey (HIPS), more than half of respondents reported recurrent bone pain, while 43% described pain severe enough to limit daily activities, and over one third required regular pharmacologic pain management. These findings align with observations from the Global HPP Registry, where fatigue and reduced mobility were among the most persistent patient-reported outcomes even after diagnosis [11].

Beyond the structural consequences of defective mineralization, accumulating biochemical evidence suggests that musculoskeletal pain in HPP arises from a convergence of local inflammatory and systemic metabolic mechanisms. At the peripheral level, enhanced formation of calcium pyrophosphate dihydrate crystals has been shown to trigger local inflammatory responses within joints and periarticular tissues, offering a plausible explanation for chronic pain in some patients. In parallel, impaired vitamin B6 metabolism resulting from deficient ALP activity may reduce neuronal availability of PLP, a cofactor essential for the synthesis of neurotransmitters involved in nociceptive modulation such as γ-aminobutyric acid (GABA) and serotonin [75,76,77,78]. This disruption could theoretically alter central pain processing and contribute to heightened pain sensitivity or reduced fatigue tolerance observed in affected individuals, although these neurochemical implications remain incompletely defined.

A complementary mechanism involves dysregulation of purinergic signaling, whereby impaired ALP-mediated conversion of pro-inflammatory ATP to anti-inflammatory adenosine leads to a sustained inflammatory milieu [77,79]. This imbalance between purinergic ligands may amplify both pain perception and systemic fatigue, particularly during physical exertion and offers a mechanistic framework for patient-reported exertional pain and delayed post-activity recovery [80]. Collectively, these mechanisms position pain in HPP as a multifactorial phenomenon arising from disordered skeletal biomechanics and altered bioenergetic signaling, in which secondary inflammatory processes may further amplify symptom intensity. Consistent with this notion, some case observations suggest that naproxen, a nonsteroidal anti-inflammatory drug, can provide partial symptomatic relief in pediatric patients, supporting the contribution of inflammatory pathways within this broader metabolic pain framework [81,82].

In the absence of ERT, this pathophysiologic cascade remains unmitigated, contributing to persistent pain, early fatigue, and reduced physical endurance.

#### 5.2.2. Growth and Skeletal Development

Growth impairment in pediatric HPP represents a subtle yet clinically meaningful aspect of disease burden that extends beyond radiographic bone pathology. Evidence from large observational cohorts of untreated children indicates that short stature is not a universal feature of HPP; however, growth impairment is particularly pronounced during infancy, most notably in those younger than two years of age. This age-dependent pattern suggests that HPP interferes with growth plate activity at a critical stage of skeletal development, when endochondral ossification and chondrocyte differentiation are most reliant on sufficient ALP activity. Infants with HPP exhibit marked deceleration in height velocity, often accompanied by modest impairment in weight gain, suggesting that growth failure in this context is primarily skeletal rather than systemic. After this early critical period, growth trajectories tend to stabilize, yet true catch-up growth is rarely observed, indicating that the potential for recovery diminishes once the growth plate architecture becomes structurally altered [41,83]. These developmental dynamics underscore that early-onset disease carries the greatest risk for persistent short stature. In many children, the reduction in height velocity during infancy results in a permanent downward shift in growth percentiles, even if subsequent progression remains proportionate. Importantly, growth restriction in HPP is predominantly longitudinal, reflecting defective mineralization at the growth plate rather than global nutritional or endocrine insufficiency. This distinction is crucial, as it positions growth failure in HPP not as a hormonal deficit but as a secondary consequence of an enzymatic block disrupting local calcium–phosphate homeostasis and hydroxyapatite deposition.

Clinical observations further reveal that height alone does not reliably indicate overall disease severity. Children with pronounced short stature tend to exhibit greater skeletal deformities and delayed motor milestones, whereas those of normal height may still present with significant dental or musculoskeletal symptoms [41]. Such variability highlights the heterogeneity of pediatric HPP and cautions against using stature as a surrogate for disease control or treatment need. For clinicians, subtle deceleration in linear growth, particularly during infancy, should serve as an early clinical signal warranting investigation of serum ALP activity and consideration of HPP in the differential diagnosis. Although growth trajectories tend to stabilize beyond early childhood, prediction of final height remains uncertain, as conventional markers of skeletal maturity may not be reliable in HPP.

While growth impairment in early childhood reflects disrupted mineralization at the growth plate, clinical experience suggests that these alterations may improve functionally when ERT is initiated early. Radiologic studies in treated children have shown that skeletal abnormalities, particularly rickets-like lesions and cortical thinning, improve within the first six months of asfotase alfa administration and that these improvements can be sustained over years of therapy. This restoration of skeletal architecture likely parallels the reactivation of normal endochondral ossification, reinforcing the concept that timely intervention not only stabilizes growth but also re-establishes the physiological dynamics of bone development [58].

Although longitudinal data remain limited, early initiation of asfotase alfa appears to stabilize growth trajectories and support the maintenance of physiological skeletal development. Continued monitoring of height velocity and skeletal morphology is therefore essential to assess long-term outcomes and optimize treatment timing in affected children.

#### 5.2.3. Functional Limitations and Health-Related Quality of Life in Pediatric HPP

The clinical expression of pediatric HPP extends far beyond impaired mineralization, influencing multiple domains of daily function and psychosocial well-being. Defective bone strength and chronic skeletal pain contribute to limitations in mobility, endurance, and participation in normal childhood activities. These findings portray HPP not only as a disorder of skeletal biology but as a chronic condition that substantially compromises health-related quality of life (HRQoL), particularly in the absence of timely ERT.

In untreated populations, limitations in mobility and self-care are among the most frequently reported consequences of chronic bone hypomineralization. Data from patient-reported outcomes highlight that these restrictions affect not only physical activity but also social participation, autonomy, and overall HRQoL. Between one third and three quarters of respondents described limitations in moderate activities such as walking, standing from a seated position, or lifting objects. Many also reported difficulty engaging in age-appropriate daily tasks, including school attendance and recreational participation [11]. Importantly, these data were collected from untreated individuals, underscoring the progressive deterioration in physical functioning that can occur in the absence of ERT. Over a five-year observation period, approximately two-thirds of respondents reported worsening of at least one symptom, suggesting that the burden of pediatric HPP intensifies over time when left untreated. Beyond physical limitations, emotional and psychosocial challenges also play a significant role. Approximately one third of surveyed children and adolescents indicated that emotional difficulties interfered with their ability to perform daily activities. As disease progression restricts independence and mobility, the resulting frustration, isolation, and loss of self-efficacy further compound the burden on both patients and their families. Despite the absence of formalized HRQoL endpoints in most clinical trials, converging data from long-term observational studies and patient registries suggest that ERT with asfotase alfa yields meaningful improvements in functional autonomy and daily living performance. Gains in motor endurance [58,84], respiratory independence [63], and self-care capacity reported in treated cohorts collectively imply a broader amelioration of physical well-being. However, these improvements have largely been inferred from functional and caregiver-reported outcomes rather than from standardized HRQoL instruments, limiting the ability to objectively quantify the psychosocial dimension of recovery. Qualitative data from post-approval follow-up indicate that families perceive not only physical recovery but also enhanced social participation and emotional stability in children receiving early treatment. Yet, the current evidence base remains inherently limited by heterogeneity in study design and the absence of disease-specific tools sensitive to the developmental context of pediatric HPP. Conventional HRQoL scales, often adapted from other metabolic or skeletal disorders, may underestimate the multidimensional impact of the disease, particularly its effects on fatigue, self-image, and cognitive-emotional development. Taken together, these findings emphasize that the functional burden of HPP extends beyond the skeleton. Beyond musculoskeletal and psychosocial limitations, gastrointestinal and metabolic manifestations may further contribute to the overall reduction in quality of life. Feeding difficulties, recurrent vomiting, and poor appetite are frequently reported in severely affected infants, sometimes necessitating enteral or PEG tube feeding to maintain adequate nutrition and growth [85]. Although the precise pathophysiology remains incompletely defined, TNSALP is expressed in the intestinal mucosa, where it dephosphorylates luminal substrates such as bacterial lipopolysaccharides (LPS) and phosphate-containing lipids. This enzymatic activity contributes to intestinal barrier defense and the detoxification of proinflammatory compounds. In HPP, deficient TNSALP activity may therefore impair mucosal protection, disrupt phosphate absorption, and alter the gut microbiome, mechanisms that could plausibly contribute to systemic inflammation or subtle nutritional imbalance [86,87,88,89].

Future research should therefore integrate patient-centered outcomes and validated psychometric measures into longitudinal follow-up protocols to more accurately define the full therapeutic benefit of asfotase alfa and to inform holistic models of care that extend beyond skeletal restoration.

## 6. Risk–Benefit Profile and Safety Considerations of Ert

The clinical safety profile of asfotase alfa has remained favorable over more than a decade of therapeutic use, with most adverse events being mild and manageable. Injection-site reactions are the most common, occurring in approximately two thirds of patients, typically presenting as transient erythema, pain, or pruritus [43]. Systemic hypersensitivity reactions are rare, and anaphylaxis has been reported only in isolated cases [51]. To date, no new systemic safety signals have been reported during longitudinal follow-up, and there is no evidence of cumulative renal, hepatic, or cardiovascular toxicity attributable to asfotase alfa. However, formal long-term safety studies addressing potential organ-specific effects remain limited, underscoring the importance of ongoing pharmacovigilance and registry-based monitoring.

A more complex and clinically relevant aspect of asfotase alfa safety relates to its immunogenic potential. Longitudinal data from clinical trials and registry cohorts indicate that the majority of treated patients develop anti-asfotase alfa antibodies, with neutralizing activity reported in a substantial proportion of seropositive cases [63]. The median time to antibody detection is typically within the first six months of therapy. Despite this high seroconversion rate, most studies have found no consistent association between antibody development and adverse events, immune-mediated reactions, or treatment discontinuation [58]. Clinical efficacy does not appear to be systematically affected by antibody positivity, and biochemical and radiographic improvements have been observed across pediatric cohorts irrespective of serologic status during the first year of therapy [90]. However, emerging observations from longer-term longitudinal studies suggest that, in a subset of patients, particularly those with more severe baseline disease or prolonged treatment exposure, higher antibody titers and neutralizing activity may coincide with delayed or incomplete radiographic response. While these findings do not establish causality, they raise the possibility that immunogenicity may contribute to interindividual variability in treatment outcomes. This possibility is illustrated by an isolated case report describing progressive attenuation of therapeutic effect after 2.5 years of continuous treatment in a patient with high-titer neutralizing antibodies, in whom temporary treatment interruption combined with immune tolerance induction successfully restored response [91]. It is noteworthy that antibody testing for asfotase alfa is not commercially available, limiting routine screening for neutralizing antibody formation in clinical practice.

By analogy with other enzyme replacement therapies, severe genotypes and early exposure to high antigenic loads may predispose to higher immunogenicity, but such associations remain unconfirmed in HPP [92]. Larger longitudinal registries and mechanistic studies are therefore needed to clarify whether antibody development reflects disease severity, genetic background, or cumulative treatment duration. From a broader perspective, the safety profile of asfotase alfa should be interpreted in relation to its therapeutic benefit. Compared with historical symptomatic interventions, which often carried metabolic or renal risks without addressing the underlying enzymatic defect, its adverse effects are generally mild, predictable, and manageable. The persistence of clinical benefit despite antibody formation supports the overall durability of ERT, while reinforcing the need for continued long-term safety surveillance.

## 7. The Need for Systematic Screening in Pediatric HPP

Early recognition of HPP remains a formidable obstacle, not only because of clinical and biochemical heterogeneity, but also due to the absence of standardized screening protocols in pediatric practice. In this context, recent efforts to develop neonatal and pediatric-age screening strategies mark a potential paradigm shift. In a large-scale population-based study from Japan, Kumamoto University screened 45,632 newborns by measuring TNAP activity in dried blood spots (DBSs), demonstrating that high-throughput newborn screening (NBS) for HPP is feasible at population scale and may identify affected infants well before clinical manifestations emerge [93].

This proof of concept underscores a crucial point: by the time overt symptoms such as skeletal abnormalities, fractures, dental loss, or growth delay [31] become apparent, much of the disease burden may already be established, narrowing the window for optimal therapeutic intervention. Population-based screening would allow early diagnosis, timely initiation of ERT, and potentially prevention of irreversible damage.

Importantly, the Japanese TNAP-based screening approach demonstrated the ability to identify not only infants with life-threatening perinatal or severe infantile HPP, but also individuals with milder phenotypes and even carriers of pathogenic variants. While some cases of severe perinatal HPP were recognized prenatally based on radiographic abnormalities, the majority of affected infants benefited from early biochemical detection, enabling pre-symptomatic diagnosis and structured clinical monitoring. This model illustrates how screening can substantially reduce the diagnostic latency that frequently characterizes non-classical forms of HPP.

Beyond its immediate clinical utility, this screening program holds considerable epidemiological and translational significance for the field of pediatric HPP. Early identification of both severe and attenuated forms of the disease facilitates the establishment of well-characterized patient cohorts, enabling more robust genotype–phenotype correlation studies and contributing to mutation databases specific to diverse populations. Such data are essential not only for improving diagnostic precision but also for development of individualized treatment strategies [94,95,96]. Moreover, the systematic follow-up of screen-positive cases offers a unique opportunity to delineate the natural history of pediatric HPP and assess long-term outcomes of early therapeutic intervention. Although the feasibility of implementing similar programs internationally would depend on factors such as local disease prevalence and healthcare resources, the Japanese model represents a compelling proof of concept that could reshape early diagnostic paradigms and improve outcomes for children affected by this rare but serious disorder.

## 8. Limitations and Future Perspectives in Pediatric HPP

### 8.1. Molecular Determinants of Therapeutic Variability in Pediatric HPP

Historically, HPP has been classified according to the age at symptom onset into perinatal, perinatal benign, infantile, childhood, adult, and odonto-HPP forms. Although this clinical classification remains useful in routine practice, accumulating evidence suggests that these categories represent arbitrary divisions within a continuous disease spectrum, as considerable variability exists both within and across age groups. Moreover, age-based classification does not adequately capture the dynamic evolution of HPP throughout life or reflect the broad phenotypic continuum associated with different *ALPL* variants. Consequently, recent studies have shifted the emphasis from the timing of clinical presentation toward the underlying molecular architecture of the disease, recognizing genotype as a determinant of disease severity and long-term clinical expression [97]. This conceptual evolution has refined the interpretation of genotype–phenotype relationships by demonstrating that the clinical expression of HPP is more closely related to the functional consequences and inheritance pattern of *ALPL* variants than to age at onset alone [31,98]. Consistent with this evolving understanding, severe HPP is generally considered a rare autosomal recessive disorder, whereas moderate disease may be inherited through either recessive or dominant mechanisms, and mild HPP is recognized as a common dominantly inherited condition [99].

Beyond allelic configuration, the distribution of individual *ALPL* variants also exhibits marked geographic variability. Data from the Global HPP Registry identified c.571G>A (p.Glu191Lys), c.1250A>G (p.Asn417Ser), and c.1559del as the most prevalent pathogenic variants worldwide, although their frequencies differ substantially across populations. The c.571G>A variant predominates in European cohorts, whereas c.1559del is particularly enriched in Japanese patients, in whom biallelic pathogenic variants are also observed more frequently than in other populations. Furthermore, studies conducted in Japanese cohorts have shown that homozygosity represents an important determinant of disease severity, and both homozygous and compound heterozygous genotypes are strongly associated with life-threatening perinatal and infantile HPP [99]. Such variability likely reflects founder effects and genetic drift, underscoring that the mutational landscape of HPP is not globally uniform. Consequently, genotype–phenotype correlations derived from one population cannot be assumed to apply universally, and therapeutic data generated in genetically heterogeneous cohorts may obscure mutation-specific effects.

Importantly, the clinical implications of genetic testing extend beyond variant identification alone. In selected clinical settings, particularly prenatal evaluation or early infancy, the detection of well-characterized biallelic *ALPL* genotypes may improve early risk stratification. Genotypes such as c.[1559delT];[1559delT] and c.[1001G>A];[1001G>A] have consistently been associated with life-threatening perinatal or infantile HPP [100,101]. However, biallelic inheritance should not be interpreted as a universal surrogate marker of disease severity, as clinically attenuated forms have also been described in patients harboring two pathogenic *ALPL* variants, highlighting the persistent complexity of genotype–phenotype relationships [102]. This distinction has important implications for precision medicine, emphasizing that risk stratification should rely on comprehensive genotype interpretation rather than variant count alone.

Although genotype has emerged as an important determinant of disease severity, its role in predicting response to ERT remains considerably less well defined. To date, no robust genotype-specific differences in the efficacy of asfotase alfa have been demonstrated. Current evidence therefore supports the use of genotype primarily for diagnostic confirmation, genetic counseling, and prognostic assessment, while therapeutic decisions continue to rely on an integrated evaluation of clinical manifestations and biochemical abnormalities [103]. Whether specific *ALPL* variants influence long-term therapeutic outcomes remains an important area for future investigation.

This discrepancy reflects the persistent challenge of translating genetic findings into clinically actionable therapeutic information. Despite the transformative impact of ERT, the molecular architecture of pediatric HPP continues to impose fundamental constraints on therapeutic precision. At the core of this complexity lies the allelic heterogeneity of the *ALPL* gene. While truncating variants (nonsense and frameshift mutations) typically lead to markedly reduced or absent TNSALP activity and are frequently associated with severe perinatal or infantile phenotypes, the majority of reported variants are missense substitutions with highly variable structural and catalytic consequences. The functional effect of these substitutions depends on their localization within critical regions of the enzyme, including the catalytic site, crown domain, metal-binding environment, or homodimeric interface [104,105]. However, for many variants, detailed structural and kinetic characterization remains incomplete, limiting the integration of genotype-specific information into therapeutic algorithms.

Beyond variant distribution, the relationship between residual TNSALP enzymatic activity and clinical expression remains only partially resolved. While extremely low in vitro activity values consistently associate with perinatal-lethal and severe infantile phenotypes, the inverse relationship lacks predictive robustness. Variants retaining moderate or even apparently substantial residual catalytic activity may still manifest with clinically significant disease [106]. This non-linearity reflects the intrinsic limitations of conventional enzymatic assays, which primarily quantify catalytic turnover under standardized laboratory conditions but do not fully capture structural instability, defective dimerization, impaired intracellular trafficking, or dominant-negative interference within the homodimeric complex. Importantly, several studies have demonstrated partial overlap in measured residual TNSALP activity between affected individuals and population controls, particularly for variants retaining more than 50% of wild-type enzymatic function [104]. This overlap limits the use of enzymatic thresholds as standalone prognostic tools. Together, these observations indicate that neither residual activity nor allele frequency, when considered in isolation, provides sufficient discriminatory power for precise pathogenic classification or therapeutic stratification.

The presence of dominant-negative mutations adds another layer of complexity. Because TNSALP functions as a homodimer, mutant subunits may disrupt wild-type partners, reducing effective enzymatic activity beyond simple haploinsufficiency. Such interactions complicate genotype interpretation based solely on allelic status and challenge assumptions that exogenous enzyme supplementation uniformly compensates for endogenous molecular dysfunction [99,107]. Whether these mutation specific structural dynamics influence long-term response variability remains insufficiently explored in genotype-stratified cohorts. Notably, genotype analyses indicate that attenuated or clinically mild forms of HPP frequently arise not from intrinsically benign variants, but from dominant-negative effects exerted by severe alleles or from compound heterozygosity combining severe and moderate variants [107]. This observation further complicates phenotype prediction, as apparent clinical mildness may reflect complex allelic interactions rather than preserved intrinsic enzymatic function.

Collectively, these considerations indicate that while asfotase alfa corrects the extracellular biochemical consequences of TNSALP deficiency, it does not eliminate the biological variability arising from distinct *ALPL* variants. Refinement of therapeutic strategies in pediatric HPP will therefore require closer integration of molecular characterization with longitudinal clinical assessment. Although genotype specific treatment algorithms are not currently implemented in routine practice, accumulating registry data and functional studies suggest that variant specific properties, including residual activity and dominant-negative behavior, may contribute to differences in long-term therapeutic stability.

### 8.2. Therapeutic Limitations and Emerging Strategies

Following the molecular complexity outlined above, several practical limitations continue to shape the long-term management of pediatric HPP and define priorities for future therapeutic development. While asfotase alfa has unequivocally improved survival and skeletal outcomes in severe perinatal and infantile forms, its mode of administration remains a significant challenge. The requirement for three to six subcutaneous injections per week is associated with frequent injection-site reactions and represents a substantial treatment burden for children and caregivers alike [11]. Over time, this burden may compromise adherence, particularly in patients requiring lifelong therapy and in those with milder phenotypes where perceived benefit may be less immediately evident. These practical constraints highlight that, even in the era of disease-modifying therapy, treatment sustainability remains an unresolved issue in pediatric HPP. An additional limitation that continues to influence outcomes in pediatric HPP is the absence of harmonized newborn screening programs, which contributes to substantial inequalities in access to timely diagnosis and care. In current practice, identification of affected children remains highly dependent on local clinical awareness, availability of biochemical testing, and national healthcare infrastructures, resulting in marked heterogeneity in diagnostic pathways across regions. This variability translates into delayed or missed diagnoses, particularly in infantile and childhood-onset forms with initially subtle manifestations and leads to inconsistent timing of treatment initiation. Such disparities may partially explain the wide variability in clinical outcomes reported in contemporary cohorts, despite the availability of effective ERT, and represent a systemic limitation that cannot be addressed by therapeutic innovation alone. Beyond diagnostic challenges, first-generation ERT raises questions regarding therapeutic optimization across the heterogeneous pediatric HPP spectrum. Current dosing regimens are largely extrapolated from pivotal trials focused on survival and radiographic outcomes in severe disease, while evidence guiding dose individualization, treatment duration, or de-escalation strategies in milder phenotypes remains limited [108]. Moreover, although asfotase alfa effectively corrects extracellular substrate accumulation and supports skeletal mineralization, it does not uniformly address all functional manifestations, particularly neuromuscular symptoms such as muscle weakness and fatigue, which significantly affect motor development and quality of life in children [41].

These limitations have driven the development of second-generation enzyme replacement therapies aimed at improving both efficacy and tolerability. Efzimfotase alfa represents a leading example of this approach, incorporating targeted molecular modifications designed to enhance catalytic activity and optimize pharmacokinetic properties while preserving bone-specific targeting. Structural changes within the TNSALP domain, removal of selected N-linked glycosylation sites, and substitution of the IgG1 Fc region with a human IgG2/4 Fc are intended to increase intrinsic enzymatic potency and support less frequent dosing. Preclinical data demonstrate enhanced enzymatic activity and effective skeletal targeting, suggesting that such optimization may reduce injection frequency without compromising therapeutic benefit [109]. For pediatric patients, particularly those diagnosed early, this evolution holds promise for improving adherence and long-term treatment acceptance. Importantly, emerging translational evidence indicates that optimized enzyme replacement may exert clinically meaningful effects beyond bone. Experimental models have shown that efzimfotase alfa improves mitochondrial respiratory capacity in skeletal muscle independently of skeletal mineralization status, highlighting a potential mechanism for addressing muscle weakness and fatigue, features that are increasingly recognized as central contributors to disease burden in pediatric HPP [110]. Early therapeutic intervention, enabled by timely diagnosis, may therefore support not only skeletal development but also neuromuscular function during critical periods of growth.

Gene-based therapeutic strategies provide an important experimental framework for future disease modification in HPP, although their clinical applicability remains limited at present. AAV8-mediated neonatal gene therapy has demonstrated sustained alkaline phosphatase expression, prolonged survival, and near-normal bone mineralization in murine models of severe infantile disease following a single intramuscular injection [111]. These findings highlight the critical importance of very early intervention, targeting a narrow developmental window in which adequate enzymatic activity appears essential for normal skeletal and neuromuscular maturation. In this context, the absence of systematic newborn screening programs represents a major barrier, as timely identification of affected neonates would be a prerequisite for any early gene-based intervention. Despite encouraging preclinical results, substantial translational challenges persist. Long-term safety, immune responses to viral vectors, durability and regulation of transgene expression, and the narrow therapeutic window for alkaline phosphatase activity remain unresolved, particularly in the setting of a growing child. The potential risk associated with unregulated or supraphysiological enzyme levels further underscores the need for precise control of expression [111]. Consequently, AAV-mediated gene therapy should currently be regarded as a proof-of-concept approach that informs future therapeutic development rather than a near-term alternative to ERT in pediatric HPP.

Taken together, the evolving therapeutic landscape of pediatric HPP reflects both substantial progress and persistent unmet needs. While ERT has transformed outcomes for many patients, limitations related to treatment burden, diagnostic delays, and heterogeneity in care delivery continue to constrain its full potential. Advances in second-generation enzyme replacement therapies offer a pragmatic path toward improving long-term tolerability and addressing non-skeletal manifestations, whereas gene-based approaches currently remain confined to the experimental domain. Ultimately, meaningful improvements in pediatric HPP outcomes will require not only therapeutic innovation but also earlier diagnosis, greater harmonization of clinical practices, and longitudinal strategies tailored to the changing needs of children across development. Integrating these elements will be essential to translate scientific advances into sustained, equitable clinical benefit.

## 9. Conclusions

The management of pediatric HPP has undergone a fundamental transformation, evolving from survival-oriented supportive care to targeted ERT and function-centered, long-term management. The introduction of asfotase alfa has markedly improved survival and skeletal outcomes in severe early-onset disease, redefining expectations for growth, mobility, and developmental recovery. As survival has become achievable, therapeutic success is now increasingly defined by sustained functional performance and health-related quality of life. Despite these advances, pediatric HPP remains a biologically and clinically complex disorder. Marked allelic heterogeneity of the *ALPL* gene, variable residual enzymatic activity, and dominant-negative effects limit the predictive value of genotype alone and constrain precise therapeutic stratification. In parallel, delayed recognition continues to restrict the full benefit of ERT during critical periods of skeletal development, underscoring the importance of early diagnosis and timely intervention. In this context, ERT should not be viewed as a standalone solution but rather as the central component of an integrated care model that includes symptom-directed management, rehabilitation, dental surveillance, and longitudinal functional assessment. Looking forward, meaningful optimization of outcomes in pediatric HPP will depend on aligning therapeutic innovation with earlier identification strategies, harmonized diagnostic pathways, and robust outcome frameworks that extend beyond radiographic and biochemical endpoints. Advances in next-generation enzyme replacement therapies and emerging gene-based approaches hold promise for improving treatment burden and addressing non-skeletal manifestations, but their potential can only be realized within systems capable of delivering early, individualized, and sustained care.

## Figures and Tables

**Table 1:** Evolution of outcome measures in pediatric HPP studies across the ERT era. The table illustrates the progressive shift from predominantly survival- and radiographic-based endpoints toward functional, patient-centered, and quality-of-life outcomes, reflecting the evolving therapeutic objectives in pediatric HPP.

Study	Year	Skeletal Outcomes	Functional/Patient-Centered Outcomes
Whyte et al. [63]	2012	RSS, RGI-C	Respiratory function
Whyte et al. [38]	2016	RGI-C	Overall survival; Respiratory function
Whyte et al. [47]	2016	RSS, RGI-C; Height, weight, BMI Z-scores	6MWT; PODCI; CHAQ
Schroth et al. [64]	2021	—	Dental assessment
Padidela et al. [65]	2025	Length/height and weight percentiles, Z-scores	Respiratory support; 6MWT; Bleck scale; BAMF; Analgesic use; PedsQL

6MWT—6 Minute Walk Test, BAMF—Brief Assessment of Motor Function, BMI—Body Mass Index, CHAQ—Child Health Assessment Questionnaire, PedsQL—Pediatric Quality of Life Inventory, PODCI—Pediatric Outcomes Data Collection Instrument, RGI-C—Radiographic Global Impression of Change, RSS—Rickets Severity Scale.

**Table 2:** Key challenges in the clinical implementation and long-term management of asfotase alfa in pediatric HPP. The table summarizes major diagnostic, therapeutic, and follow-up–related challenges discussed in the text, reflecting areas of ongoing uncertainty and variability in clinical practice rather than formal treatment recommendations.

Domain	Key Challenge	Clinical Implications
Diagnosis and patient eligibility	Heterogeneous clinical spectrum with diagnostic gray zones, particularly in non-classical or attenuated phenotypes	Delayed recognition and uncertainty regarding treatment initiation
Treatment initiation	Absence of a strict binary distinction between “mild” and “severe” disease	Need for individualized treatment decisions guided by functional burden rather than phenotype alone
Dosing strategies	Lack of validated dosing de-escalation or tapering protocols	Reliance on empirical dose adjustments in selected patients
Predictive biomarkers	Absence of reliable biomarkers predicting sustained response or safe dose modification	Limited ability to individualize treatment intensity based on laboratory parameters alone
Long-term follow-up	No consensus on integrating laboratory and radiologic data into adaptive treatment algorithms	Variability in follow-up strategies across clinical centers
Biochemical surveillance	Complex interpretation of ALP and PLP during ERT due to assay limitations, preanalytical artifacts, and age-related variability	Risk of misinterpretation requiring strict technical and clinical oversight
Treatment intensity	Uncertainty in defining optimal treatment intensity across the clinical spectrum	Need for integrated clinical, functional, and radiologic assessment
Treatment adherence	Chronic treatment burden and uncertainty regarding perceived benefit	Increased risk of reduced adherence over time
Outcome assessment	Transition from survival-based endpoints to functional and quality-of-life–focused outcomes	Requirement for broader, patient-centered outcome frameworks
Asymptomatic individuals	Lack of evidence-based surveillance strategies for preclinical or asymptomatic HPP	Need for balanced longitudinal monitoring without unnecessary medical intervention

## Data Availability

No new data were created or analyzed in this study.

## Abbreviations

The following abbreviations are used in this manuscript:

6MWT	6 Minute Walk Test
BAMF	Brief Assessment of Motor Function
BMI	Body Mass Index
CHAQ	Child Health Assessment Questionnaire
ERT	Enzyme Replacement Therapy
HPP	Hypophosphatasia
P10	Deca-Aspartate
PEA	Phosphoethanolamine
PedsQL	Pediatric Quality of Life Inventory
PLAP	Placental alkaline phosphatase
PLP	Pyridoxal-5 Phosphate
PODCI	Pediatric Outcomes Data Collection Instrument
PPi	Inorganic pyrophosphate
RGI-C	Radiographic Global Impression of Change
RSS	Rickets Severity Scale
TNSALP	Tissue-nonspecific alkaline phosphatase
